# Exceptional Clinical Response to BRAF-Targeted Therapy in a Patient with Metastatic Sarcoma

**DOI:** 10.7759/cureus.439

**Published:** 2015-12-28

**Authors:** Demytra Mitsis, Mateusz Opyrchal, Yujie Zhao, John M Kane III, Richard Cheney, Kilian E Salerno

**Affiliations:** 1 Medical Oncology, Roswell Park Cancer Institute; 2 Medical Oncology, Carle Foundation Hospital; 3 Surgery, Roswell Park Cancer Institute; 4 Pathology, Roswell Park Cancer Institute; 5 Radiation Medicine, Roswell Park Cancer Institute

**Keywords:** sarcoma, braf, molecular profiling, targeted therapy

## Abstract

Soft tissue sarcomas (STS) are a rare and heterogeneous group of tumors arising from mesenchymal tissue comprising 1% of all adult cancers. The prognosis of metastatic STS is dismal. As there are few active drugs, there is a critical need to find new therapeutic alternatives in order to improve outcomes.

Most sarcoma subtypes are fairly resistant to standard chemotherapy regimens and/or have a short duration of response. The era of molecular targeted therapy may present new treatment options for metastatic STS and an opportunity to drive biomarker discovery and personalized medicine.

This case report describes a patient with a synchronous metastatic high-grade sarcoma who was treated with BRAF-targeted therapy with resolution of his metastatic lesions. To our knowledge, this exceptional response has not been described in sarcoma literature.

The patient presented with a large anterior abdominopelvic wall mass. Biopsy showed a high-grade spindle cell sarcoma. Staging scans confirmed two pulmonary metastases.

He was initiated on combination chemotherapy with mixed results. He received 50 Gy of radiation to the primary tumor followed by two additional cycles of combination chemotherapy, with an interval increase in the size of the pulmonary metastases.

Molecular testing revealed a BRAF V600E mutation in the primary tumor. The patient was initiated on dabrafenib/trametinib with a dramatic response and resolution of his pulmonary metastases. The patient underwent surgical resection of his primary mass, and pathology confirmed no evidence of residual disease.

Detailed genetic characterization of STS coupled with novel therapeutic strategies, including molecular targeted therapy, may have the potential to transform the care of patients diagnosed with sarcoma.

## Introduction

The management of STS patients with synchronous metastases poses a difficult dilemma and treatment often includes multi-modality approaches. Despite elaborate treatment regimens, the poor prognosis of those with metastatic STS has changed very little over the past three decades.

Increasingly, human mesenchymal malignancies are being classified by the abnormalities that drive their pathogenesis [1]. Molecularly targeted therapies may lead to the emergence of more customized treatment options in patients with STS.

This case report depicts a patient with a large high-grade spindle cell STS with synchronous pulmonary metastases. BRAF-targeted therapy resulted in a dramatic response. The choice of therapy and dramatically observed outcome have not been reported in the literature, to our knowledge.

An improved understanding of the molecular biopsy of STS may lead to additional clinical trials testing novel therapies specifically designed to thwart critical pathways responsible for this malignancy and potentially improve survival in metastatic STS.

## Case presentation

A 69-year-old male presented with a one-year history of an enlarging abdominopelvic wall mass. He sought emergency department (ED) care for bleeding. On examination, he was found to have a large, ulcerated mass in the right lower quadrant. Informed patient consent was obtained.

**Figure 1:**
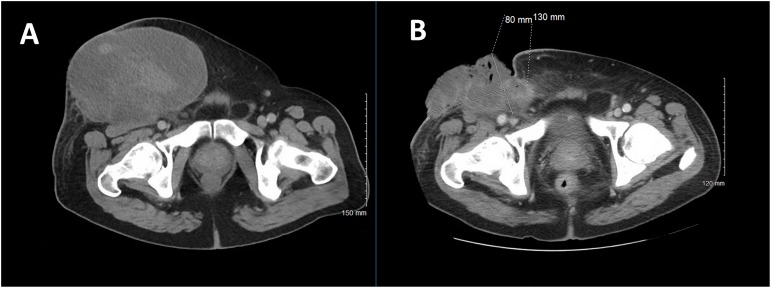
Primary STS at Diagnosis and After Chemotherapy, Plus Radiation A: CT of the abdomen and pelvis reveals a large anterior abdominopelvic mass measuring 19 x 12.8 x 16.8 cm. B: Primary mass after one cycle of chemotherapy (gemcitabine/docetaxel) and local radiation therapy (50 Gy).

Computed tomography (CT) of the abdomen and pelvis with contrast revealed a large heterogeneous mass anterior to the right inguinal region (Figure 1A). A biopsy was consistent with a high-grade sarcoma. Surgical debridement was done for bleeding and infection. He was then referred for further management.

An internal review of the pathology was consistent with a high-grade sarcoma. Histopathology showed spindle cell-like morphology with a mitotic rate of 4-8 per 10 high-power fields, frequent apoptosis, and necrosis. Immunohistochemical stains revealed tumor cells positive for vimentin and CD99 and negative for desmin, melanin-A, CD34, calretinin, CK-7, CD56, TTF-1, SMA, S100, NSE, FL-1, and both LMW and HMW cytokeratin. FISH analysis was negative for both MDM2 amplification and CHOP rearrangement.

**Figure 2:**
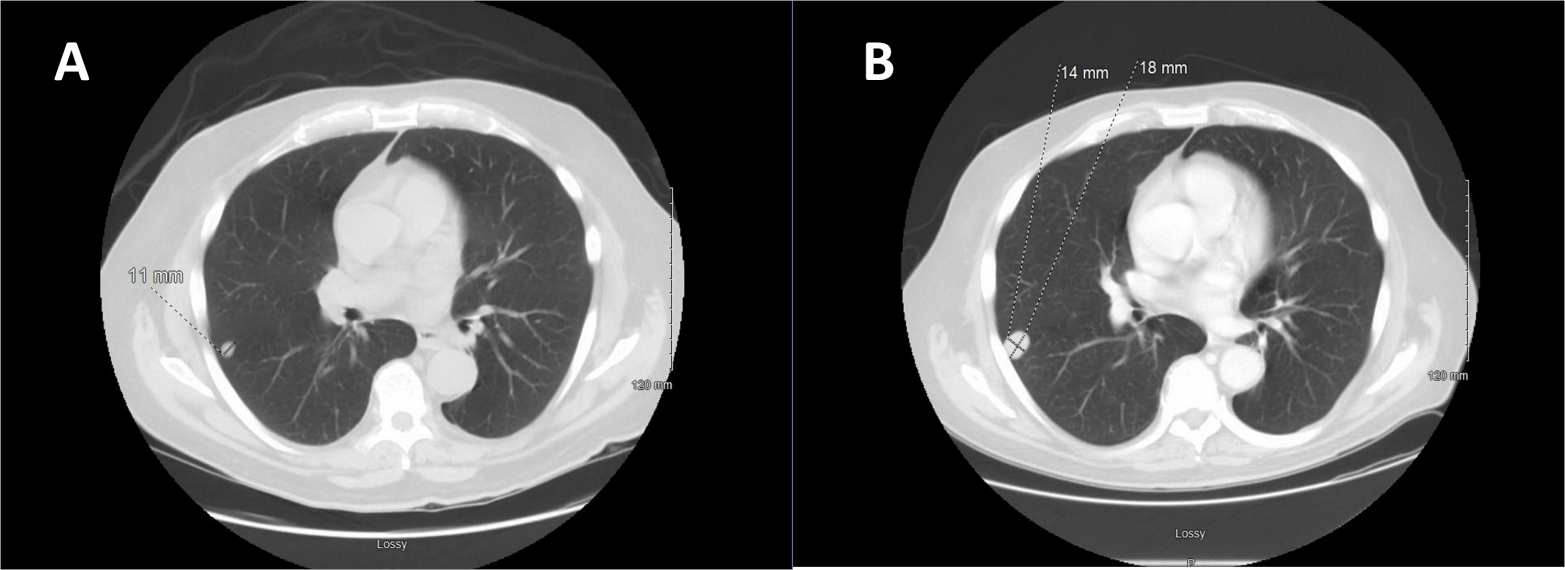
CT Chest at Diagnosis and After One Cycle of Combination Chemotherapy A: CT scan of the chest at diagnosis reveals a 1.1 cm nodule in the right lower lobe. B: CT scan of the chest after one cycle of gemcitabine/docetaxel reveals an increase in the size of the right lower lobe pulmonary nodule (1.4 x 1.8 cm).

A staging CT scan of the chest with contrast revealed two non-calcified, well-circumscribed pulmonary nodules (1.1 cm in the right lower lobe (RLL) (Figure 2A) and 1 cm in the left lower lobe (LLL)).

The patient was evaluated by the sarcoma multidisciplinary team and felt to have synchronous pulmonary metastatic disease. He was not a candidate for anthracycline-based treatment. He was initiated on chemotherapy with gemcitabine, 675 mg/m2 IV every seven days for two doses per 21-day cycle (days 1 and 8), and docetaxel, 100 mg/m2 IV once per cycle (day 8).

A restaging CT after one cycle of chemotherapy revealed mixed results. The RLL lesion was stable, but the LLL lesion had increased in size. No new lesions were seen. The abdominopelvic wall mass had decreased in size and had become necrotic. In addition, multiple pulmonary emboli and a right femoral vein thrombus were found for which enoxaparin was initiated.

**Figure 3:**
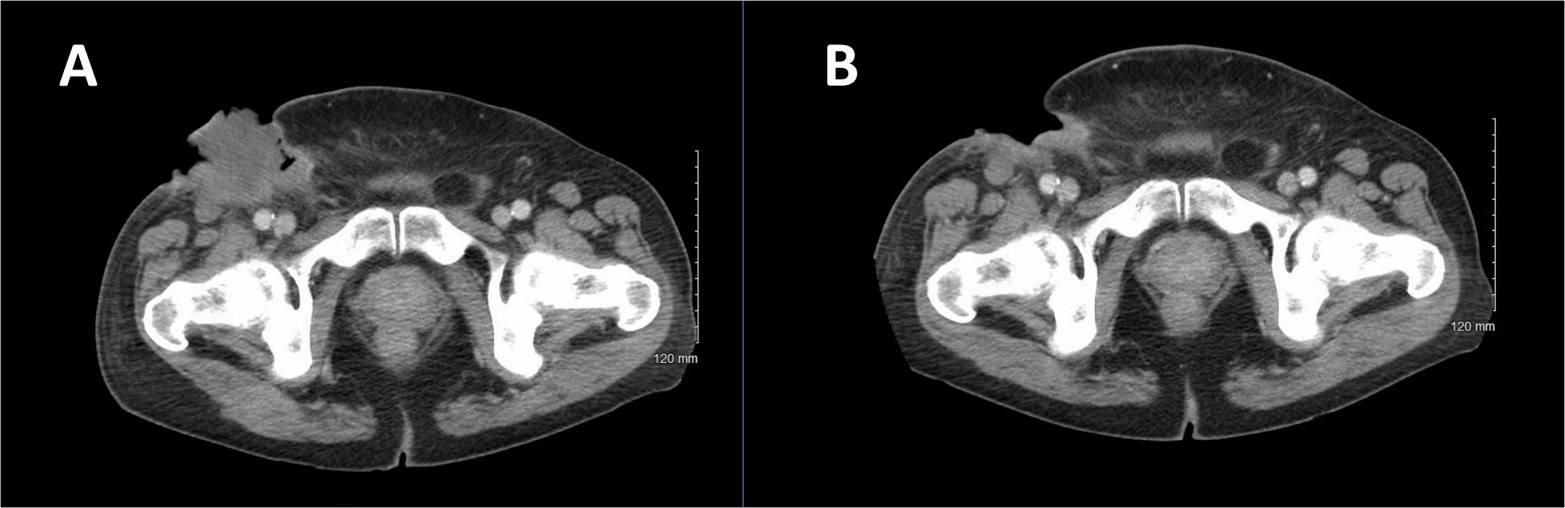
CT Scan of the Abdomen and Pelvis Before and After Six Weeks of BRAF-targeted Therapy A: CT scan of the abdomen and pelvis prior to initiation of dabrafenib/trametinib therapy. B: Regression of primary mass after six weeks of BRAF targeted therapy. Surgical excision of the remnant mass confirmed no evidence of residual sarcoma.

**Figure 4:**
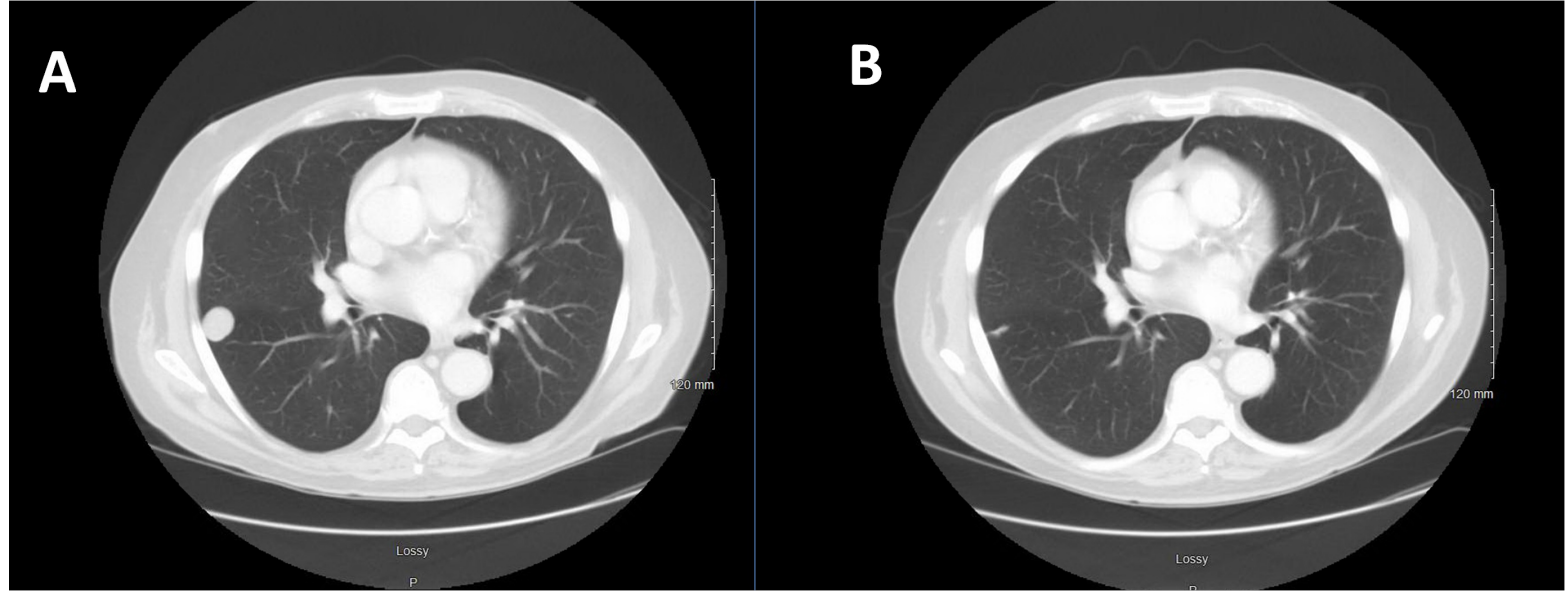
CT Chest Before and After Six Weeks of BRAF-targeted Therapy A: CT scan of the chest after receiving a total of three cycles of combination chemotherapy (gemcitabine/docetaxel) and prior to BRAF-targeted therapy. B: CT scan of the chest after six weeks of dabrafenib/trametinib therapy, revealing regression of the right pulmonary metastasis.

Subsequently, the patient developed a significant hemorrhage from his primary mass and required massive transfusion. Chemotherapy was held, and the patient received radiation therapy (RT) to a total treatment dose of 50 Gy to the primary mass for local control. Post-RT imaging revealed an overall decrease in the primary mass and enlargement of the right pulmonary nodule without any new lesions (Figures 1B, 2B). Bleeding was controlled following RT. He then completed two additional cycles of gemcitabine and docetaxel with an increase in the size of the right pulmonary metastasis (Figures 3A, 4A). 

Given his disease progression on prior chemotherapy and limited next therapeutic options, dual next-generation sequencing was performed and showed a BRAF V600E mutation. The patient was initiated on dabrafenib, plus trametinib. This combination regimen was chosen over vemurafenib monotherapy because dabrafenib, plus trametinib, significantly improved overall survival in previously untreated patients with metastatic melanoma with *BRAF* V600E or V600K mutations, without increased overall toxicity [1]. Imaging after six weeks of treatment revealed a dramatic response in both pulmonary metastases and the primary tumor (Figures 3B, 4B). A continued imaging response was seen after 14 weeks of dabrafenib/trametinib.

Treatment was held, and the patient underwent a definitive wide resection of the primary sarcoma, including an en-bloc portion of the right external oblique muscle with sartorius muscle rotation flap and partial primary closure. Pathology showed no residual malignancy. One month post-surgery, imaging showed a complete resolution of the lung nodules. The patient is currently without evidence of disease.

## Discussion

Despite the rarity of sarcomas compared to other adult malignancies, the exploration of novel therapies is essential for improving outcomes in this difficult to treat disease. There is no data to direct the optimal management of patients with STS and limited synchronous metastases, and current National Comprehensive Cancer Network (NCCN) guidelines are non-specific about treatment options in this group of patients.  Unfortunately, there are a limited number of effective therapies. Advanced-stage patients treated with the most active drugs (anthracyclines and ifosfamide) achieve only a median survival of around one year [2]. A single institution study found no improved survival in patients with synchronous metastases treated with metastasectomy [3]. Thus, new treatment approaches are needed.

In this case, molecular gene profiling was performed with the hope of finding a targetable mutation and a potential new treatment approach. Recent studies have suggested that selecting molecularly targeted agents (MTAs) based on the molecular profile of the patient’s tumor independently of the tumor location and histology might improve patient outcomes. A study by Von Hoff, et al. compared the progression-free survival (PFS) using a treatment regimen selected by molecular profiling (MP) of a patient's tumor with the PFS for the most recent regimen on which the patient had experienced progression. Molecular targets were identified in 98% of patients. In 27% of patients, the molecular profiling approach resulted in a longer PFS on an MP-suggested regimen than on the regimen on which the patient had just experienced progression [4].

Recent advances have been made in the understanding of the molecular biology of sarcomas. Increasingly, human mesenchymal malignancies are classified by the abnormalities that drive their pathogenesis, though few are currently targeted therapeutically [5]. Systemic surveys of cancer genomes with next-generation sequencing have proven an effective approach in identifying targetable genetic alterations in specific cancer types. The list of potential targets will continue to grow with the expanded use of second-generation sequencing technologies, which detect not only genome-wide-copy-number changes, but rearrangements, insertion/deletions, and mutations. Simultaneously, new treatment options on these bases will need to be explored.

Although BRAF inhibition is a possible therapeutic option for 1% of adult GIST patients with a BRAF-V600 mutation, a dependence of STS on oncogenic Raf has not been well described [5-6]. Although BRAF mutation in sarcoma has not been systemically evaluated, a recent study examined the BRAF mutational status of 90 sarcomas. BRAF mutation was absent in all cases [7]. Another study assessed the treatment effect of vemurafenib on 122 cases of BRAF V600-mutated non-melanomas. Two of the 122 cases were sarcomas, and one patient achieved a partial response to vemurafenib [8]. 

In this unique case, molecular profiling led to the use of a systemic therapy that would not have otherwise been considered with a dramatic response. Defining how and when to integrate such therapies into clinical practice, although challenging, may lead to a paradigm shift towards more personalized therapy.

A recently presented randomized Phase II trial comparing molecularly-targeted therapy based on tumor molecular profiling versus conventional therapy in patients with refractory cancer did not demonstrate that the administration of MTAs outside their indications, according to the pre-specified treatment algorithm, improves PFS as compared to the treatment of physician choice in heavily pretreated patients [9]. Certainly, this approach warrants further investigation into the RTK/MAPK pathway and other critical pathways known to play a role in sarcomagenesis.

## Conclusions

We report a case of exceptional clinical response to BRAF-targeted therapy in a patient with metastatic sarcoma to the lung, leading to resolution of his disease. Treatment choice was based on molecular profiling of the primary tumor. 

To date, genomic characterization efforts in cancer have primarily focused on epithelial and hematologic cancers. Given the need for new therapies in sarcomas to improve outcomes and the potential for use of MTAs, as seen in this case, the inclusion of sarcomas in future studies is needed. The Cancer Genome Atlas (TCGA) project is initiating a comprehensive genomic analysis of various sarcoma subtypes. A detailed genetic characterization of these tumors is likely to benefit future understanding of the disease and contribute to novel treatment approaches [6]. In the meantime, there remains a critical need to find new therapeutic alternatives and consider molecular profiling in metastatic sarcoma refractory to traditional therapy.
